# Supplementary material for: Is Telemedicine in Primary Care a Good Option for Polish Patients with Visual Impairments Outside of a Pandemic?
Source: Int J Environ Res Public Health. 2022 May 24;19(11):6357. doi: 10.3390/ijerph19116357 (PMC9180207; doi:10.3390/ijerph19116357)
Supplement: Supplementary file 1 [file ijerph-19-06357-s001.zip › ijerph-1658120-supplementary.pdf]

Table S1: Frequency of the PCP's services utilization in the past six months.

	Never		Once		Two times		3-5 times		≥6		Total	
	N	%	N	%	N	%	N	%	N	%	N	%
**Level of visual impairment**												
The poor-sightedness	22	14.4	15	9.8	39	25.5	52	34.0	25	16.3	153	71.2
The blindness	17	27.5	9	14.5	15	24.2	12	19.4	9	14.5	62	28.8
**Sex in the poor-sighted group**												
Poor-sighted women	12	12.4	12	12.4	21	21.6	33	34.0	19	19.6	97	63.4
Poor-sighted men	10	17.9	3	5.4	18	32.1	19	33.9	6	10.7	56	36.6
**Sex in the blind group**												
Blind women	8	29.7	3	11.1	7	25.9	6	22.2	3	11.1	27	43.5
Blind men	9	25.8	6	17.1	8	22.9	6	17.1	6	17.1	35	56.5
**Additional disability**												
Yes	12	14.1	9	10.6	18	21.2	27	31.8	19	22.4	85	39.5
No	27	20.8	15	11.5	36	27.7	37	28.5	15	11.5	130	60.5
**Chronic disease**[1]												
Yes	18	13.2	13	9.6	29	21.3	48	35.3	28	20.6	136	63.3
No	21	26.6	11	13.9	25	31.7	16	20.2	6	7.6	79	36.7

[1] $chi^2=17.013$; $p=0.002$.

Table S2: The utilization of the computer and Internet by the study participants in percentages.

	Computer using				Internet using			
	Yes		No		Yes		No	
	N	%	N	%	N	%	N	%
**Level of visual impairment**								
The poor-sightedness	125	80.1	31	19.9	132	84.6	24	15.4
The blindness	54	85.7	9	14.3	57	90.5	6	9.5
Total	189	86.3	30	13.7	189	86.3	6	9.5
**Sex in the poor-sighted group**								
Women	75	76.5	23	23.5	80	81.6	18	18.4
Men	50	86.2	8	13.8	52	89.7	6	10.3
**Sex in the blind group**								
Women	21	75.0	7	25.0	24	85.7	4	14.3
Men	33	94.3	2	5.7[1]	33	94.3	2	5.7
**Age**								
18-39 years	66	97.1	2	2.9	68	100.0	-	-
40-59 years	66	93.4	7	9.6	71	97.3	2	2.7
60 and more	45	59.2	31	40.8[2]	48	63.2	28	36.8[5]
**Level of education**								
Primary education	21	56.8	16	43.2	21	56.8	16	43.2
Secondary education	81	82.6	17	17.4	87	88.8	11	11.2
Higher education	77	91.7	7	8.3[3]	81	96.4	3	3.6[6]
**Place of residence**								
Rural	30	96.8	1	3.2	31	100.0	-	-
Urban to 50 k inhabitants	38	84.4	7	15.6	41	91.1	4	8.9
Urban from 50 k to 100 k inhabitants	21	87.5	3	12.5	23	95.8	1	4.2
Urban from 100 k to 500 k inhabitants	37	80.4	9	19.6	38	82.6	8	17.4
Urban over 500 k inhabitants	53	72.6	20	27.4[4]	56	76.7	17	23.3[7]

[1] $chi^2=4.725$; $p=0.030$; [2] $chi^2=39.916$; $p=0.000$; [3] $chi^2=21.069$; $p=0.000$; [4] $chi^2=9.582$; $p=0.048$; [5] $chi^2=52.235$; $p=0.000$; [6] $chi^2=35.114$; $p=0.000$; [7] $chi^2=13.854$; $p=0.008$.

Table S3: The level of the computer skills among the study group.

	Lack		Low		Average		High	
	N	%	N	%	N	%	N	%
**Level of visual impairment**								
The poor-sightedness	23	14.7	15	9.6	65	41.7	53	34.0
The blindness	7	11.1	6	9.5	26	41.3	24	38.1
Total	30	13.7	21	9.6	91	41.6	77	35.1
**Sex in the poor-sighted group**								
Women	18	18.4	10	10.2	39	39.8	31	31.6
Men	5	8.6	5	8.6	26	44.9	22	37.9
**Sex in the blind group**								
Women	5	17.9	2	7.1	11	39.3	10	35.7
Men	2	5.7	4	11.4	15	42.9	14	40.0
**Age[1]**								
18-39 years	-	-	4	5.9	26	38.2	38	55.9
40-59 years	3	4.1	6	8.2	33	45.2	31	42.5
60 and more	27	35.5	10	13.2	31	40.8	8	10.5
**Level of education[2]**								
Primary education	16	43.2	1	2.7	18	48.7	2	5.4
Secondary education	10	10.2	15	15.3	46	46.9	27	27.6
Higher education	4	4.8	5	6.0	27	32.1	48	57.1
**Place of residence**								
Rural	-	-	4	12.9	14	45.2	13	41.9
Urban to 50 k inhabitants	3	6.7	4	8.9	18	40.0	20	44.4
Urban from 50 k to 100 k inhabitants	3	12.5	1	4.2	13	54.1	7	29.2
Urban from 100 k to 500 k inhabitants	6	13.0	5	10.9	21	45.7	14	30.4
Urban over 500 k inhabitants	18	24.7	7	9.6	25	34.2	23	31.5

[1]$chi^2=65.198$; $p=0.000$; [2]$chi^2=61.074$; $p=0.000$.

Table S4: The level of the Internet skills among the study group.

	Lack		Low		Average		High	
	N	%	N	%	N	%	N	%
**Level of visual impairment**								
The poor-sightedness	23	14.7	7	4.5	62	39.8	64	41.0
The blindness	5	7.9	9	14.3	23	36.5	26	41.3
Total	28	12.8	16	7.3	85	38.8	90	41.1
**The poor-sighted group**								
Women	17	17.3	5	5.1	39	39.8	37	37.8
Men	6	10.3	2	3.4	23	39.7	27	46.6
**The blind group**								
Women	4	14.3	3	10.7	10	35.7	11	39.3
Men	1	2.9	6	17.1	13	37.1	15	42.9
**Age**[1]								
18-39 years	-	-	-	-	20	29.4	48	70.6
40-59 years	1	1.4	5	6.9	33	45.1	34	46.6
60 and more	27	35.5	10	13.2	31	40.8	8	10.5
**Level of education**[2]								
Primary education	16	43.2	2	5.4	17	46.0	2	5.4
Secondary education	9	9.2	10	10.2	45	45.9	34	34.7
Higher education	3	3.6	4	4.8	23	24.4	53	64.2
**Place of residence**								
Rural	-	-	-	-	15	48.4	16	51.6
Urban to 50 k inhabitants	3	6.7	2	4.4	16	35.6	24	53.4
Urban from 50 k to 100 k inhabitants	1	4.2	4	16.7	9	37.5	10	41.6
Urban from 100 k to 500 k inhabitants	7	15.2	5	10.9	19	41.3	15	32.6
Urban over 500 k inhabitants	17	23.3	5	6.8	26	35.6	25	34.3

[1]chi^2=89.740; p=0.000; [2]chi^2=63.498; p=0.000; [3] chi^2=24.901; p=0.015.

Table S5: Representative quotes for the needs of PVIs using TCs.

Exact date and time of TC	*"An important element in the use of TC by people with reduced visual perception or who are blind is the timeliness of the scheduled advice (the registrar arranges for the patient to call at 2 p.m., the patient and his assistant wait for the phone call, and the doctor contacts at 5 p.m., when the assistant is no longer there, or the patient is busy with other activities and cannot use the aids he has prepared - notebook, dictaphone, etc.)."*<br><br>*"That the TC takes place at a fixed time (or at an approximate time) - so I can take care of privacy and better prepare for the conversation (to write down my questions and doctor's recommendations)."*
Long-enough time for the visit	*"Adequate time because it can sometimes be difficult to read the results on my own and the stress of rushing doesn't make it easier."*<br><br>*"Sometimes a little more time is needed, e.g. to write something down, it is important that the TC does not put too much time pressure."*<br><br>*"Above all, the doctor's attitude, understanding that a little more time is needed to handle the technology."*
Psychosocial competencies of the staff	*"To simply be treated as a subject and not as an object."*
Medical staff competencies related to the functioning of PwDs	*"That the doctor should be aware that he is talking to a blind person and be able to give him comprehensive information."*<br><br>*"Knowledge of the subject of disability, often doctors are surprised that a blind person lives independently, etc."*
Well-designed TC	*"Their availability, at least the phone option."*<br><br>*"Short wait times for TC."*<br><br>*"For the doctor to receive my test results prior to TC and review them before calling me, so that I don't have to walk to pick up the results and then read them to the doctor during the TC."*<br><br>*"So the doctor calls (not the patient)."*
Awereness of the patient's health situation	*"Teleconsultation to a doctor who knows my medical history."*
Technological issues	*"Openness of doctors to issue e-prescriptions and e-referrals, which a blind person can read independently."*<br><br>*"That the TC would be available to everyone, i.e. it would not be via some platform that I cannot handle."*<br><br>*"It would be good if e-prescriptions were introduced everywhere; it often happens that even though I use the TC, I still have to go in person to the clinic and collect the prescription. It would be much easier if codes for e-prescriptions were sent by text message or e-mail."*
